# Effects of nitrogen fertilizer basal-to-top-dressing ratios on maize straw decomposition, soil carbon and nitrogen, and bacterial community structure in different soil textures on the north china plain

**DOI:** 10.3389/fmicb.2025.1506155

**Published:** 2025-02-05

**Authors:** Jingyu Li, Xiaonan Yang, Rui Hou, Yujie Ma, Yanqun Wang, Yang Ma, Wenchao Zhen, Yuanyuan Huang, Xin Fu, Zhengping Peng, Mingxin Men

**Affiliations:** ^1^National Key Laboratory of Crop Improvement and Regulation in North China, Hebei Agricultural University, Baoding, China; ^2^College of Resources and Environmental Sciences, College of Land and Resources, Key Laboratory of Farmland Ecological Environment of Hebei Province, Hebei Agricultural University, Baoding, China; ^3^Biology Institute, Hebei Academy of Sciences, Shijiazhuang, China

**Keywords:** loam and clay loam, N fertilizer basal-to-top-dressing ratios, maize straw decomposition, SOC, TN, soil microbial diversity

## Abstract

Straw return is a recognized agricultural practice that improves soil quality, reduces reliance on chemical fertilizers, and supports sustainable agriculture. Its effectiveness is influenced by microbial changes under varying soil properties and fertilization practices. In a wheat–maize rotation system, field experiments were conducted over 2 years in loam and clay loam soils with five fertilizer (N) application treatments (i.e., no N fertilizer (N0) and N fertilizer basal-to-top-dressing ratios of 3:7 (N3:7), 4:6 (N4:6), 5:5 (N5:5), and 6:4 (N6:4)) to investigate the dynamics of maize straw decomposition, changes in soil organic carbon (SOC) and total nitrogen (TN) concentrations, soil bacterial diversity and abundance, and their interactions. Our results showed that the optimization of N fertilizer basal-to-top-dressing ratios enhanced SOC and TN by accelerating maize straw decomposition and nutrient release, as well as increasing plant carbon and nitrogen inputs. At the wheat maturity stage, the decomposition rate of maize straw reached 69.48–75.04%. The N4:6 and N5:5 ratios exhibited higher decomposition rates and C and N release rates in both soil textures. Compared to N0, N application treatments increased SOC and TN concentrations by 7.90–14.17% and 7.94–33.60%, respectively. The effects were most pronounced with the N4:6 ratio in loam and the N5:5 ratio in clay loam. Both soil textures had the same dominant bacterial phyla, but species abundance differed significantly. Loam had a significantly higher relative abundance of Proteobacteria and lower relative abundances of Gemmatimonadetes, Actinobacteria, and Chloroflexi than clay loam. N application significantly influenced bacterial diversity, with higher diversity observed with the N4:6 ratio in loam and the N5:5 ratio in clay loam. Structural equation modeling indicated that different N application treatments in loam influenced maize straw decomposition by altering the soil C/N ratio and bacterial community diversity, while in clay loam, N application treatments influenced maize straw decomposition mainly by altering the soil C/N ratio. Overall, the N4:6 treatment in loam and the N5:5 treatment in clay loam accelerated the decomposition and nutrient release of maize straw, enhanced SOC, TN, and bacterial community abundance, and provided a scientific basis for efficient straw utilization and sustainable agricultural development in the North China Plain region.

## 1 Introduction

China produces approximately 772 million tons of crop straw annually, with maize straw accounting for 34% of total straw resources (Yang et al., 2023). However, direct burning of straw severely pollutes the atmospheric environment, whereas returning straw to the field not only alleviates environmental pressure but also significantly improves soil fertility and crop yield (Liu J. et al., 2023; Palmieri et al., 2017). Numerous studies have shown that straw return can enhance soil physical and chemical properties, by reducing bulk density, improving aggregate structure, increasing porosity, and boosting concentrations of SOC, TN, and total phosphorus (TP), thus increasing the grain yield of crops (Chen et al., 2017; Chen et al., 2014; Chen et al., 2016; Tian et al., 2015). Straw decomposition is a process of gradual nutrient release and continuous changes in the C/N ratio, which may affect the soil nutrient supply. Soil texture significantly affects straw decomposition owing to differences in aeration, nutrient retention, and water-holding capacity. For example, studies have shown that straw decomposition rates are significantly and negatively correlated with soil clay content (Lei et al., 2022; Thomsen et al., 2003), whereas other studies indicate that decomposition is faster in fine-textured soils under drought conditions (Li et al., 2020). Straw return is often combined with N fertilizer application to adjust the soil C/N ratio and alleviate N limitation. One meta-analysis revealed that increasing N application significantly enhanced initial straw decomposition rates but had no significant effect during the later stages of decomposition (Min et al., 2022).

Soil microorganisms play crucial roles in straw decomposition (Bastida et al., 2006; Chen et al., 2014; Zhang et al., 2018). The greater the number of microorganisms, the faster the straw decomposes (Bastida et al., 2006). Soil texture is the second most influential factor affecting soil microorganisms, followed by pH (Xia et al., 2020). Fungi are more closely related to soil texture than bacteria, and fungal alpha diversity indices are positively correlated with sand content. As the clay content increases, the relative abundances of certain fungi (e.g., Basidiomycota and Ascomycota) and filamentous bacteria (e.g., Actinobacteria and Chloroflexi) significantly increase (Xia et al., 2020). Additionally, in agroecosystems, the metabolism of soil heterotrophic microorganisms is limited by soil nutrients such as C, N, and P (Wang et al., 2024). The combined application of N fertilizer after straw return positively affects microbial diversity and abundance (Song et al., 2024). N application directly affects soil bacterial diversity and indirectly influences bacterial community abundance, thereby promoting straw decomposition (Zeng et al., 2016).

The North China Plain is a major grain-producing region in China, where straw returning has recently been widely adopted to boost crop yields (Peng et al., 2017; Yang et al., 2019). However, the effectiveness of this practice is often hindered by management practices and soil conditions (Chen et al., 2017; Min et al., 2022). Improper straw return can lead to incomplete decomposition, low utilization efficiency, poor carbon and nitrogen sequestration, and ultimately reduced crop yields. The basal-to-top-dressing ratios of N fertilizer significantly affect straw decomposition and soil carbon and nitrogen sequestration (Min et al., 2022; Zhu et al., 2018). This optimal ratio varies widely across regions and even within the same region, depending on soil conditions (Huang et al., 2016; Smith et al., 2007). In the North China region, the primary soil textures are loam and clay loam. Despite this, there is a lack of studies on how the basal-to-top-dressing ratios of N fertilizer affect maize straw decomposition, soil carbon and nitrogen transformation, and their interactions with microorganisms in these soil textures.

Based on a 2-year field experiment conducted in the North China Plain region, our study aimed to investigate the impact of varying N fertilizer basal-to-top-dressing ratios on maize straw decomposition, nutrient release, and changes in SOC and TN concentrations across different soil textures within wheat–maize rotation systems. We also examined how these N application treatments influenced the soil microbial communities and their relationships with SOC and TN. Our hypotheses were two-fold. First, we expected that N application would accelerate straw decomposition and nutrient release, leading to increased SOC and TN concentrations, whereas the optimal basal-to-top-dressing ratios of N fertilizer were expected to differ in the two soil textures. Second, we anticipated that the characteristics of straw decomposition and changes in SOC and TN concentrations would be significantly affected by the soil microbial communities. Overall, our findings are expected to provide valuable insights for optimizing straw resource utilization and advancing sustainable agricultural practices in the North China Plain region.

## 2 Materials and methods

### 2.1 Experimental materials

**Figure 1:**
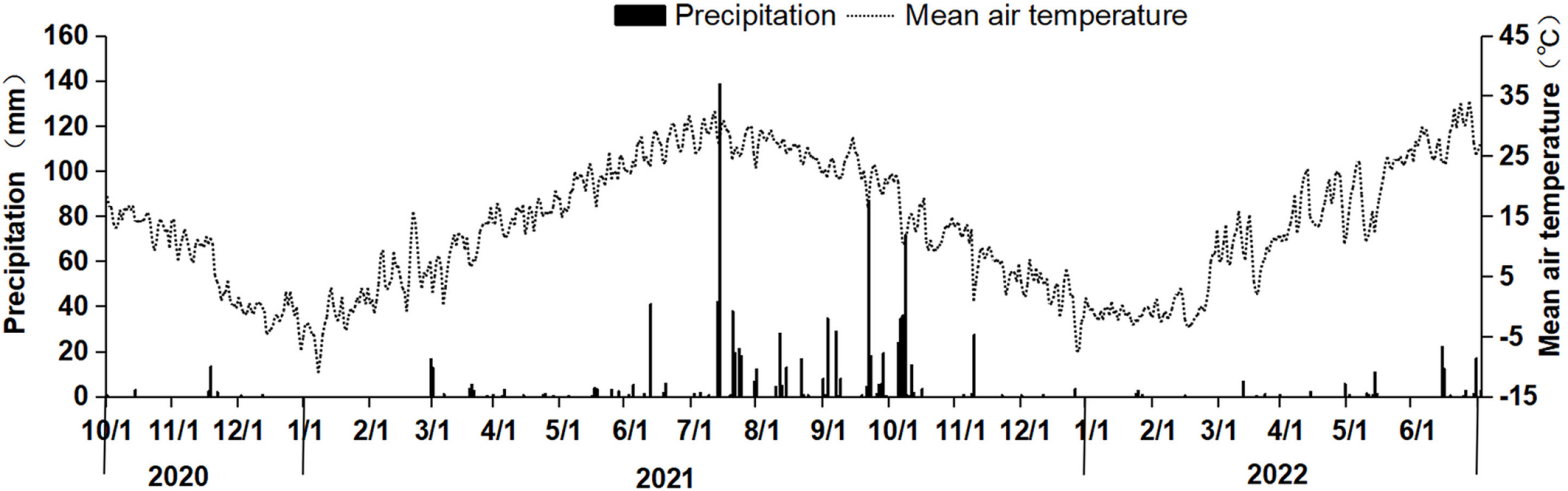
Daily precipitation and mean air temperature during the straw decomposition period of maize in the wheat–maize rotation system.

The experiment was conducted in Bai Mu Village, Jiajiakou Town, Ningjin County, Hebei Province (37°36′51″N, 115°07′23″E). The experimental site is approximately 19 m above sea level and has a temperate continental monsoon climate, with an annual average temperature of 12.8°C and an annual average precipitation of 449.1 mm. The predominant cropping system is a winter wheat–summer maize rotation, with two crops per year. The wheat variety used in this study was Gaoyou 2018. The fertilizers tested included urea (46% N), single superphosphate (16% P₂O₅), diammonium phosphate (15% N, 42% P₂O₅), and potassium chloride (60% K₂O). The temperature and precipitation during the experiment are shown in Figure 1.

The basic physical and chemical properties of the 0–20 cm soil layer and the soil particle composition before sowing are listed in Table 1. Soil texture classification was based on the soil particle composition according to international standards.

**Table 1:** Basic physical and chemical properties of the tested soils.

Soil texture	pH	BD (g·cm^−3^)	SOC (g·kg^−1^)	TN (g·kg^−1^)	NH_4_^+^-N (g·kg^−1^)	NO_3_^−^-N (g·kg^−1^)	AP (g·kg^−1^)	AK (g·kg^−1^)	Particle composition (%)		
									Clay	Silt	Sand
Loam	8.45	1.57	11.45	1.14	2.37	22.57	13.49	143.45	7.23	62.82	29.95
Clay loam	8.44	1.59	14.20	1.35	2.42	36.92	17.20	156.40	18.00	58.65	23.35

BD, bulk density; SOC, soil organic carbon; TN, total nitrogen; AN, available nitrogen; AP, available phosphorus; AK, available potassium.

### 2.2 Experimental design

The experiment was conducted in October 2019 and included two soil textures: loam and clay loam. The following five N application treatments were applied: (i) no N fertilizer (N0) and N fertilizer basal-to-top-dressing ratios of (ii) 3:7 (N3:7), (iii) 4:6 (N4:6), (iv) 5:5 (N5:5), and (v) 6:4 (N6:4). These ratios were randomly assigned to plots of both soil textures, with three replicates, totaling 30 plots, each with an area of 48 m^2^. In each N application treatment, a total of 240 kg N·hm^−2^ was applied during the entire wheat-growing period, distributed according to the different basal-to-top-dressing ratios. The N fertilizers were applied in two stages: as a basal application at sowing and as a top-dressing during the wheat jointing stage. During the jointing stage, urea was uniformly spread on the soil surface and immediately irrigated to ensure even incorporation. Phosphorus and potassium fertilizers were applied as a one-time base application, with P_2_O_5_ and K_2_O applied at 135 kg·hm^−2^ and 105 kg·hm^−2^, respectively. After the wheat harvest on June 10, 2020, summer maize was sown using the same fertilizer application. A mixed fertilizer (600 kg·hm^−2^) containing 225 kg N, 120 kg P_2_O_5_, and 150 kg K_2_O per hectare was used. Following the maize harvest on September 30, 2020, the maize straw was crushed and returned to the field, and the plots were managed according to five N application treatment schemes beginning in 2019. Wheat was sown on October 11, 2020. In each plot, micro-plots of approximately 0.7 m^2^ were established between wheat rows, containing nylon mesh bags filled with maize straw. Nylon mesh bags were buried in the soil on October 13, 2020. After the wheat harvest on June 10, 2021, maize was planted according to local farming practices. Owing to a week of heavy rainfall, the sowing of winter wheat was delayed, which was sown on October 26, 2021. After sowing the wheat, nylon mesh bags containing maize straw were buried again between the rows of wheat. Field sampling ended after the winter wheat harvest on June 8, 2022.

The nylon mesh bag procedure was as follows. When the maize reached maturity, straw was collected from the surface, kernels were removed, and the straw was crushed into approximately 2 cm pieces using a straw crusher. The crushed straw was then dried at 75°C. We weighed 25.0 g of dried straw and placed this in a 200-mesh nylon mesh bag (9 cm × 14 cm), which was sealed using a sealing machine. The sealed nylon mesh bags were buried at a depth of 15 cm in the wheat rows on 12 October 2020, and 28 October 2021. Each plot contained 11 bags placed at an angle, spaced evenly without overlapping, and marked for identification. Field sampling was completed on 8 June 2021, and 10 June 2022. All measurements of the indicators involved in this experiment were completed by December 2023.

### 2.3 Sampling and measurement

#### 2.3.1 Maize straw decomposition rate

For the period of 2020–2021, nylon mesh bags containing maize straw were collected at 10, 20, 30, 50, 70, 130, 160, 190, 210, 230, and 240 days (wheat harvest stage) after burial. For the period 2021–2022, samples were collected at 10, 20, 30, 50, 70, 100, 140, 170, 190, 210, and 220 days (wheat harvest stage). After collection, the samples were cleaned to remove surface soil and debris and dried at 70°C to a constant weight. The weight of the remaining maize straw was recorded, and the maize straw decomposition rate was calculated.

#### 2.3.2 Soil samples

For the period of 2020–2021, soil samples were collected from a depth of 0–20 cm at 0, 20, 50, 130, 190, 210, and 240 days after straw burial. For the period 2021–2022, soil samples were collected at 0, 20, 50, 100, 140, 190, and 220 days. The soil samples were air-dried in the laboratory for the measurement of SOC and TN concentrations. The SOC concentration was measured using the potassium dichromate volumetric method (Zhang et al., 2015), and TN concentration was measured using H_2_SO_4_ digestion and a chemical analyzer (Smartchem 200). At 220 days after straw burial (wheat harvest stage) in 2021–2022, fresh soil samples from the 0–20-cm layer were collected to determine the bacterial community structure. For bacterial community structure analysis, DNA was extracted from soil samples using the MOBIO Soil DNA Extraction Kit (PowerSoil® DNA Isolation Kit). PCR amplification was performed with bacterial 16S rRNA gene V3–V4 region primers (5′-ACTCCTACGGGAGGCAGCAG-3′ and 5′-GGACTACHVGGGTWTCTAAT-3′). PCR products were analyzed by 2% agarose gel electrophoresis, and target fragments were recovered using the AxyPrep™ DNA Gel Extraction Kit. Quantitative detection of the PCR products was performed using Quantiflur-ST. Based on the sequencing requirements for each sample, the PCR amplification products were combined in equimolar amounts and analyzed using the Illumina MiSeq PE300 platform (Liu A. et al., 2022).

#### 2.3.3 Decomposition rate, release rate of C and N, and plant carbon and nitrogen inputs

Calculation-related indicators, including the decomposition rate of maize straw (Equations 1, 2), release rates of C and N (Equations 3, 4), and plant carbon and nitrogen inputs (Equations 5–10), were performed following established scientific procedures and standards, as follows:


(1) $$\mathrm{Decomposition rate}\;\left(\%\right)=\frac{M_{0}–M_{t}}{M_{0}}\times 100$$

(2) $$\mathrm{Average decomposition rate}\;\left(g\cdot d^{-1}\right)=\frac{M_{t}–M_{t-1}}{T_{t}–T_{t-1}}$$

(3) $$\mathrm{Straw}\;C\;\mathrm{release rate}\;\left(\%\right)=\frac{M_{0}C_{0}–M_{t}C_{t}}{M_{0}C_{0}}\times 100$$

(4) $$\mathrm{Straw}\;N\;\mathrm{release rate}\;\left(\%\right)=\frac{M_{0}N_{0}–M_{t}N_{t}}{M_{0}N_{0}}\times 100$$

where M_o_ is the initial dry weight of straw (g), M_t_ is the dry weight of straw at time t (g), T_t_ is the days straw was buried in the soil at time t (d), C_0_ is the initial C concentration of straw (g·kg^−1^), C_t_ is the C concentration of straw at time t (g·kg^−1^), N_0_ is the initial N concentration of straw (g·kg^−1^), and N_t_ is the N concentration of straw at time t (g·kg^−1^) (Latifmanesh et al., 2020).


(5) PCI=PCImaize+PCIwheat

(6) $$\mathrm{PCImaize}=\left\{\begin{array}{l} \left(\mathrm{Ygrain}+\mathrm{Ystraw}\right)\times \frac{26\%}{74\%}\\ \times \mathrm{Croot}+\mathrm{Ystraw}\times \mathrm{Cstraw} \end{array}\right\}\times \left(1-0.14\right)$$

(7) $$\mathrm{PCIwheat}=\left\{\begin{array}{l} \left(\mathrm{Ygrain}+\mathrm{Ystraw}\right)\times \frac{30\%}{70\%}\\ \times \mathrm{Croot}+\mathrm{Ystraw}\times \mathrm{Cstraw} \end{array}\right\}\times \left(1-0.14\right)$$

(8) PNI=PNImaize+PNIwheat

(9) $$\mathrm{PNImaize}=\left\{\begin{array}{l} \left(\mathrm{Ygrain}+\mathrm{Ystraw}\right)\times \frac{26\%}{74\%}\\ \times \mathrm{Nroot}+\mathrm{Ystraw}\times \mathrm{Nstraw} \end{array}\right\}\times \left(1-0.14\right)$$

(10) $$\mathrm{PNIwheat}=\left\{\begin{array}{l} \left(\mathrm{Ygrain}+\mathrm{Ystraw}\right)\times \frac{30\%}{70\%}\\ \times \mathrm{Nroot}+\mathrm{Ystraw}\times \mathrm{Nstraw} \end{array}\right\}\times \left(1-0.14\right)$$

where PCI is the average annual plant carbon input (Mg·hm^−2^·year^−1^); PCI maize is the carbon input from maize (Mg·hm^−2^·year^−1^); PCI wheat is the carbon input from wheat (Mg·hm^−2^·year^−1^); Ygrain is the grain yield (kg·hm^−2^); Ystraw is the straw yield (kg·hm^−2^); 26%/74 and 30%/70% are the relative proportions of root biomass to aboveground biomass for maize and wheat, respectively (Guo et al., 2019; Liang et al., 2019); Croot is the organic carbon concentration in roots (%); Cstraw is the organic carbon concentration in straw (%); PNI is the average annual plant nitrogen input (Mg·hm^−2^·year^−1^); PNI maize is the nitrogen input from maize (Mg·hm^−2^·year^−1^); PNI wheat is the nitrogen input from wheat (Mg·hm^−2^·year^−1^); N root is the TN concentration in roots (%); N straw is the TN concentration in straw (%); and 14% is the average moisture content of air-dried plant samples.

#### 2.3.4 Data analysis

Data processing and visualization were performed using Microsoft Excel 2021, Origin 2021, and R (version 4.4.1) software. Statistical analyses, including analysis of variance (ANOVA), were conducted using SPSS software (version 26.0) with Duncan’s multiple range test to test differences. The alpha diversity indices were calculated using the operational taxonomic units (OTUs) table in QIIME2 and visualized as box plots. Beta (*β*) diversity was accessed using Bray–Curtis distance matrices, and the differences in communities between treatments were analyzed using analysis of similarities (ANOSIM). Principal coordinate analysis (PCoA) and Mantel tests were performed using the BioCloud tools^1^. Structural equation models (SEMs) were also used to reveal the direct and indirect effects of different nitrogen application treatments on the straw decomposition rates in the loam and clay loam soils using the “lavaan” R package.

## 3 Results

### 3.1 Straw decomposition rate

**Figure 2:**
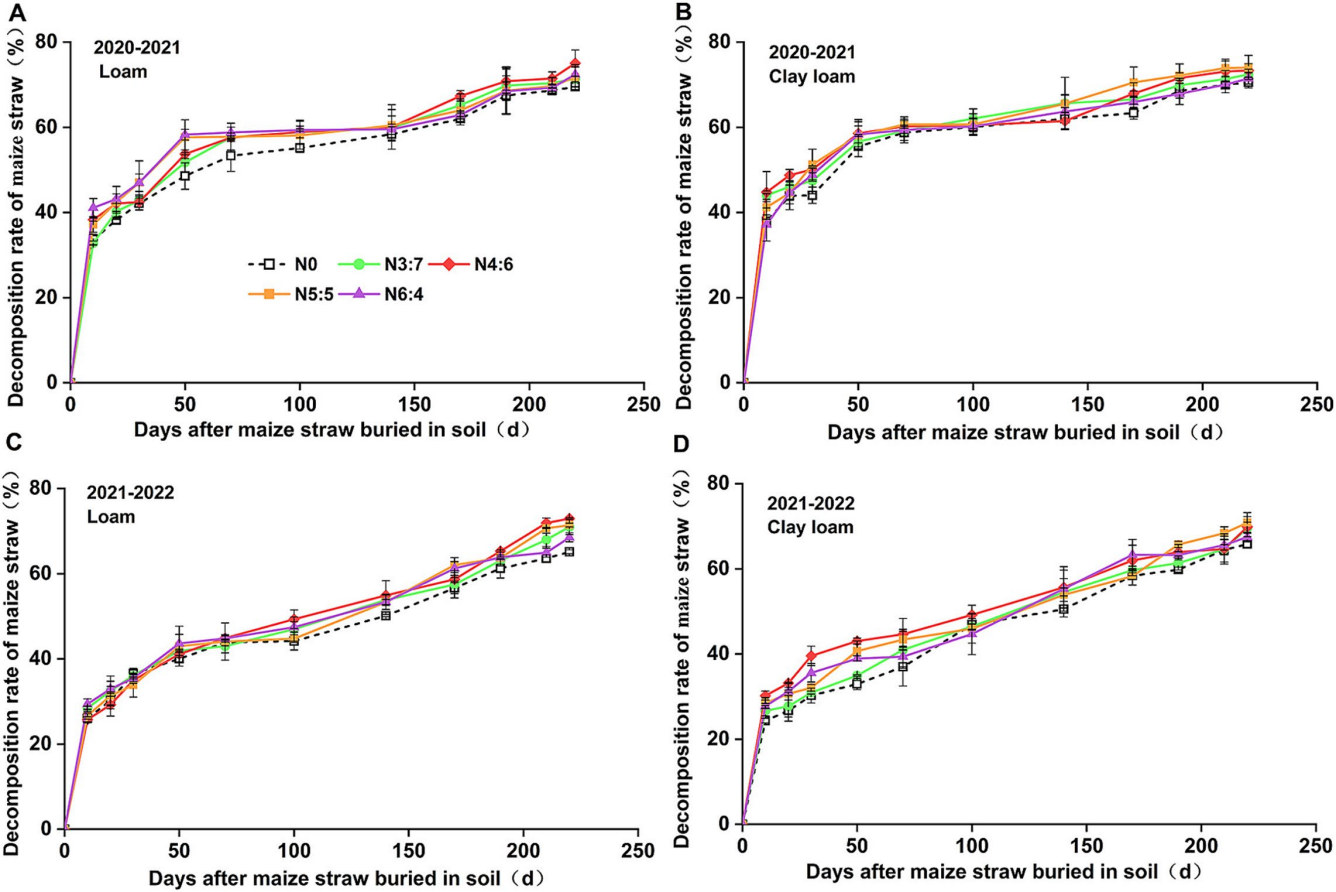
Effect of N treatments on the cumulative decomposition rate of maize straw in loam **(A,C)** and clay loam **(B,D)** from 2020 to 2022.

The results from the two-year period (Figure 2) showed that during the entire wheat-growing season, the straw decomposition rate initially increased rapidly and then slowed down. From day 0–50, straw decomposed rapidly, with decomposition rates reaching 32.92–58.57% and an average decomposition rate of 0.16–0.29 g·d^−1^. At the wheat harvest stage, the cumulative decomposition rate of straw was 69.48–75.04%, with an average decomposition rate of approximately 0.03 g·d^−1^. The average decomposition rate during the first 50 days was 9.7 times higher than that during the following 190 days.

Straw decomposition rate varied with soil texture and N fertilizer basal-to-top-dressing ratio. During the rapid decomposition phase (0–50 days), the decomposition rate of straw in loam soil increased with the N fertilizer basal-to-top-dressing ratio; the N6:4 ratio showed a 1.09–15.16% higher decomposition rate than that of the other treatments. In the clay loam soil, the straw decomposition rate first increased and then decreased with increasing N fertilizer basal-to-top-dressing ratio, with the N4:6 ratio showing a 5.70–30.62% (*p* < 0.05) higher decomposition rate than that of the other treatments. In the slow decomposition phase (after 50 days), the straw decomposition rate in both soil textures first increased and then decreased with increasing N fertilizer basal-to-top-dressing ratio. In the loam soil, the N4:6 treatment showed a 2.70–8.00% (*p* > 0.05) higher decomposition rate than that of the other treatments; whereas, in the clay loam soil, the N5:5 ratio showed a 0.96–4.99% (*p* > 0.05) higher decomposition rate than that of the other treatments. At the wheat harvest stage, the decomposition rate of straw with N application treatments increased by 3.62–8.00% (*p* < 0.05) than that with no N application treatment. The effect of soil texture on maize straw decomposition varied by year. In 2020–2021, the decomposition rate in the clay loam soil was significantly higher than that in the loam soil; whereas, in 2021–2022, the loam soil had a higher decomposition rate than the clay loam soil during the early decomposition phase (0–50 days); however, there was no significant difference during the late decomposition phase (after 50 days).

### 3.2 C and N release characteristics of straw

**Figure 3:**
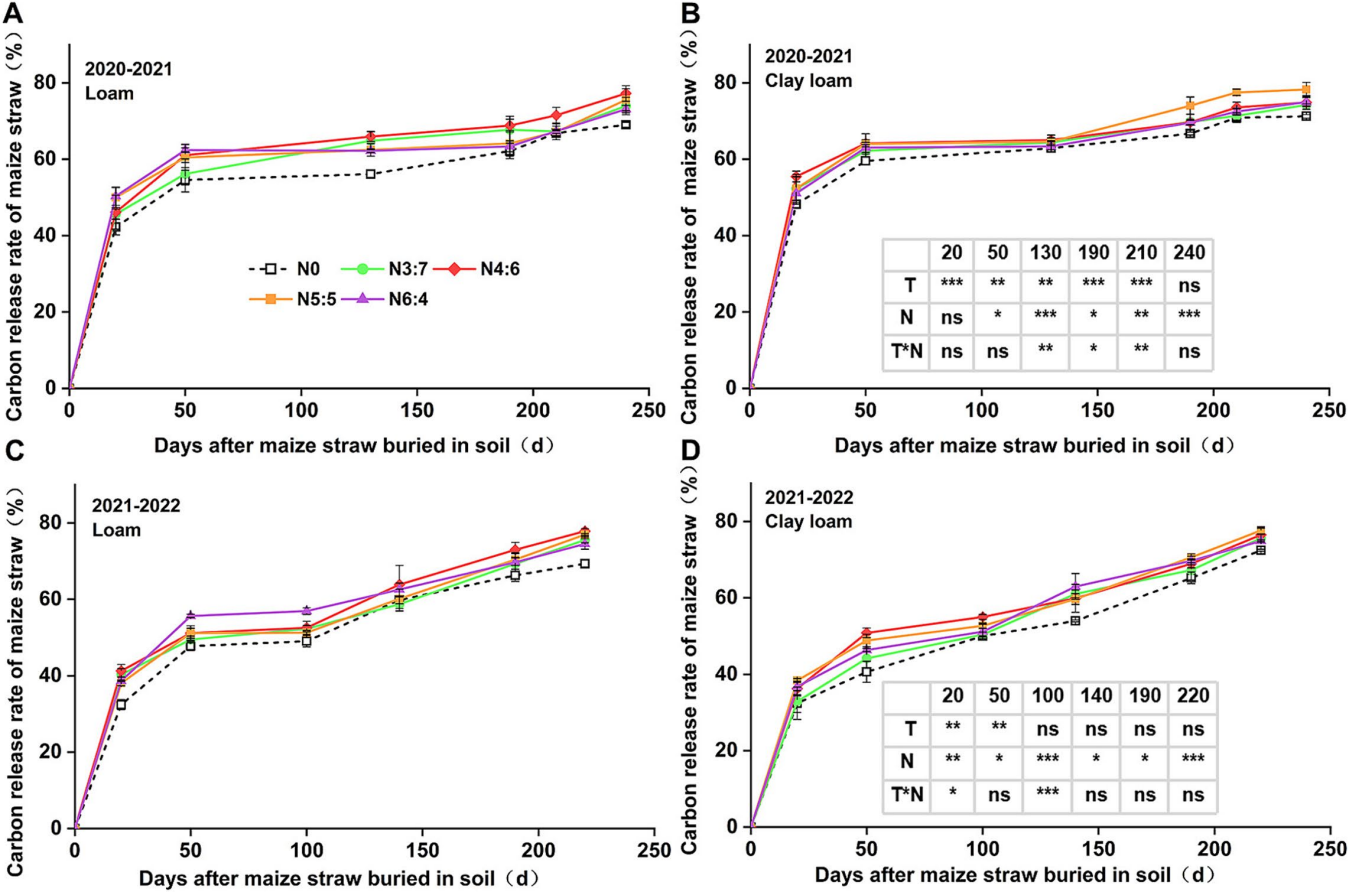
Effect of N application treatments on the cumulative C release rate of straw in loam **(A,C)** and clay loam **(B,D)** from 2020 to 2022. The table on the right side of the figure shows the significance analysis for the corresponding years. “T” and “N” represent the influences of soil texture and N treatments, respectively, based on two-way ANOVA. ns, *, **, and *** represent non-significant, *p* < 0.05, *p* < 0.01, and *p* < 0.001, respectively.

**Figure 4:**
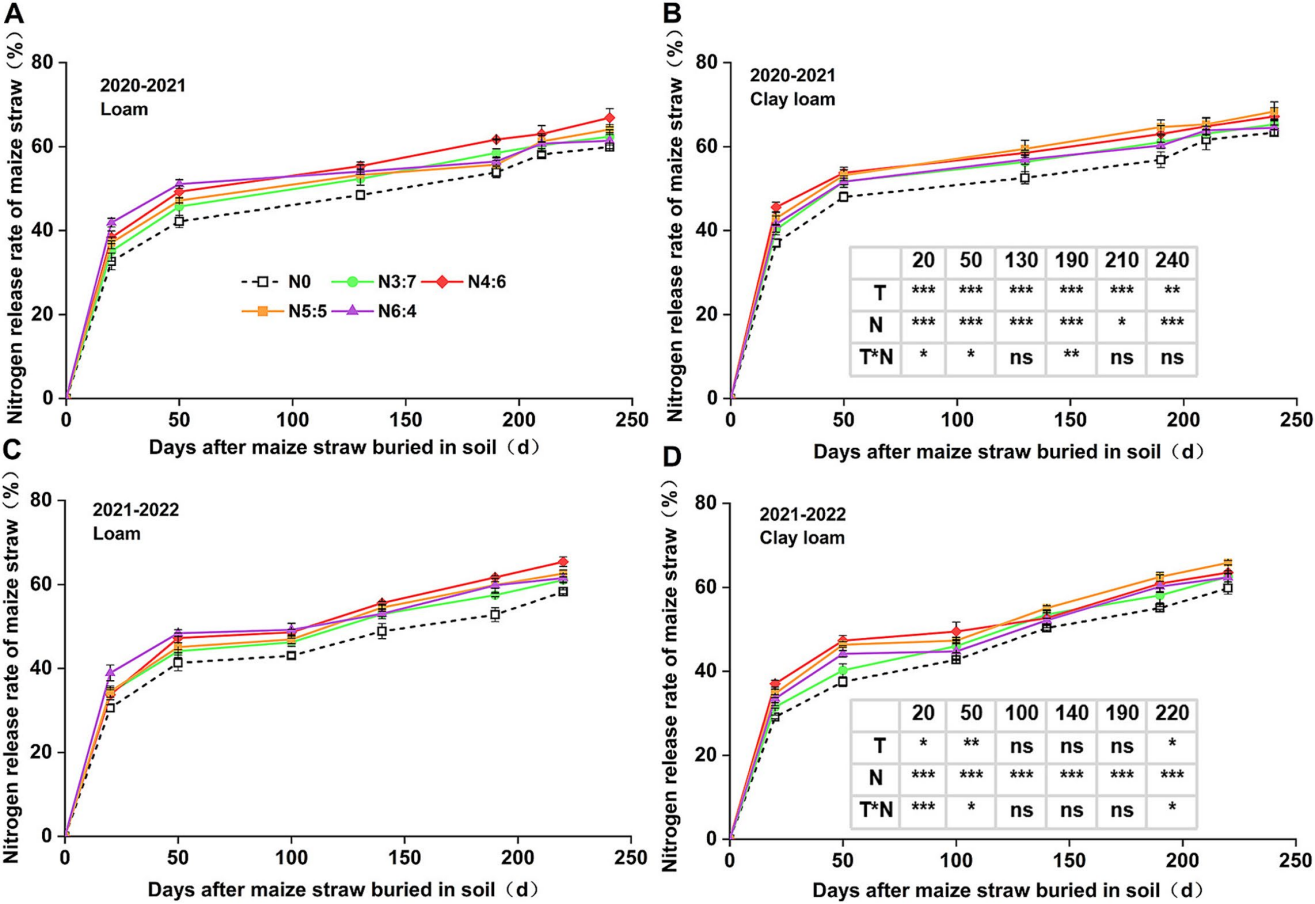
Effect of N application treatments on the cumulative N release rate of straw in loam **(A,C)** and clay loam **(B,D)** from 2020 to 2022. The table on the right side of the figure shows the significance analysis for the corresponding years. “T” and “N” represent the influences of soil texture and N treatments, respectively, based on two-way ANOVA. ns, *, **, and *** represent non-significant, *p* < 0.05, *p* < 0.01, and *p* < 0.001, respectively.

The cumulative C and N release rates from the straw (Figures 3, 4) were generally consistent with the straw decomposition rate. At 50 days, the cumulative C release rate of straw in 2020–2021 (54.59–64.16%) was significantly higher than that in 2021–2022 (40.64–55.68%). In 2020–2021, the cumulative C release rate in clay loam soil was significantly higher than that in loam soil (except at 240 days of decomposition), while in 2021–2022, the cumulative C release rate in the loam soil was significantly higher than that in the clay loam soil during the early decomposition phase (0–50 days), with no difference during the later decomposition phase (after 50 days). N fertilizer basal-to-top-dressing ratios significantly affected C release throughout the wheat-growing season, except at 20 days of decomposition in 2020–2021. During the early decomposition phase, the highest C release rate was observed for the N6:4 ratio in the loam soil and the N4:6 ratio in the clay loam soil. At the wheat harvest stage, the cumulative C release rate of straw reached 69.03–78.25%, with no significant difference observed between the two soil textures. Compared to the N0 treatment, other N application treatments increased the cumulative C release rate of straw by 3.41–12.29%, with the N4:6 treatment in the loam soil showing the highest increase of 12.29% (*p* < 0.05) and the N5:5 treatment in the clay loam soil showing the highest increase of 9.84% (*p* < 0.05).

In 2020–2021, soil texture significantly influenced the cumulative N release rate of straw. However, in 2021–2022, soil texture significantly influenced N release only during the early decomposition phase (0–50 days). N fertilizer basal-to-top-dressing ratios had a significant impact on cumulative N release from straw throughout the wheat-growing season. Similar to the cumulative C release rate, the cumulative N release rate of straw decomposition was higher in N application treatments than in those without. In the early decomposition phase (0–50 days), the cumulative release rate of N with the N6:4 ratio in the loam soil was significantly higher than that with the N3:7 ratio. At 20 days, the cumulative release rate of N with the N6:4 ratio in the clay loam soil was significantly higher than that with the N3:7 and N6:4 ratios. At the wheat harvest stage, cumulative N release rates in the loam and clay loam soils were similar, ranging from 58.25 to 68.35%. In the loam soil, the N4:6 treatment showed the highest cumulative N release rate, with a 12.22% increase (*p* < 0.05) compared to other N application treatments. In the clay loam soil, the N5:5 treatment had the highest cumulative N release rate, with a maximum increase of 10.15% (*p* < 0.05) compared to other N application treatments.

### 3.3 SOC

Both in 2020–2021 and 2021–2022, the SOC in both textures showed an “M-shaped” trend with the extension of straw decomposition time (Table 2). Soil texture and N application significantly affected SOC. During the straw decomposition process, SOC peaked at 20 and 190 days for both soil textures. In the early decomposition phase (0–50 days), the highest SOC was observed in the loam soil with an N6:4 ratio. At day 20, the SOC under the N6:4 treatment was significantly higher, by 5.89%, compared to the N3:7 treatment in 2020–2021, and by 6.61% (*p* < 0.05) compared to the N5:5 treatment in 2021–2022.

**Table 2:** Effects of N fertilizer basal-to-top-dressing ratios on SOC in loam and clay loam soils from 2020 to 2022 (g·kg^−1^).

Soil texture (T)	N application (N)	2020–2021							2021–2022						
		0d	20d	50d	130d	190d	210d	240d	0d	20d	50d	100d	140d	190d	220d
Loam	N0	11.05b	12.51c	11.90c	12.70b	14.33b	13.63b	11.90b	10.40c	12.52b	12.16b	11.63c	12.72b	13.10c	11.94c
	N3:7	11.65b	13.29b	12.54bc	12.69b	14.47b	14.43a	12.84a	11.32b	12.92ab	12.18b	12.36b	13.23a	13.22bc	12.49bc
	N4:6	11.52b	13.99a	13.24ab	13.81a	16.18a	15.01a	13.28a	12.07a	12.86ab	12.68a	12.52ab	13.32a	13.92a	13.24a
	N5:5	12.34a	13.59ab	12.32b	12.98b	15.74a	14.38a	13.02a	12.14a	12.56b	12.29b	12.08bc	12.51b	13.45abc	12.85ab
	N6:4	12.57a	14.08a	12.79a	13.27ab	15.77a	14.71a	13.25a	11.88a	13.39a	12.60a	13.02a	13.25a	13.72ab	12.96ab
Clay loam	N0	14.05a	16.25c	13.61c	16.16b	17.05b	15.29c	16.18c	13.79b	15.23b	15.13b	15.25b	14.63a	14.85d	14.40c
	N3:7	14.43a	17.27a	14.25bc	16.25b	17.50b	15.75b	16.58bc	14.08ab	15.43b	15.26ab	15.94ab	15.40ab	16.48ab	15.82ab
	N4:6	14.35a	16.97ab	13.72c	16.37ab	18.08a	15.96b	17.07ab	14.59a	16.28a	15.80ab	15.92ab	15.38ab	16.17b	16.11ab
	N5:5	14.42a	17.37a	15.07a	17.05a	18.42a	16.56a	17.45a	14.65a	16.56a	16.06a	16.55a	15.85a	16.75a	16.44a
	N6:4	14.19a	16.63bc	14.51ab	16.35ab	18.18a	15.95b	17.32a	14.09ab	15.11b	15.68ab	15.96ab	15.57a	15.45c	15.51bc
Soil texture (T)		***	***	***	***	***	***	***	***	***	***	***	***	***	***
N application (N)		***	***	***	*	***	***	***	***	**	**	**	**	***	***
T × N		**	**	***	*	*	*	ns	*	***	ns	*	*	***	ns

Mean with ANOVA results (*n* = 3). Different lowercase letters in each column represent significant differences between different N treatments (*p* < 0.05; Duncan test). “T” and “N” represent the influences of soil texture and N treatments, respectively, based on two-way ANOVA. ns, *, **, and *** represent non-significant, *p* < 0.05, *p* < 0.01, and *p* < 0.001, respectively.

**Figure 5:**
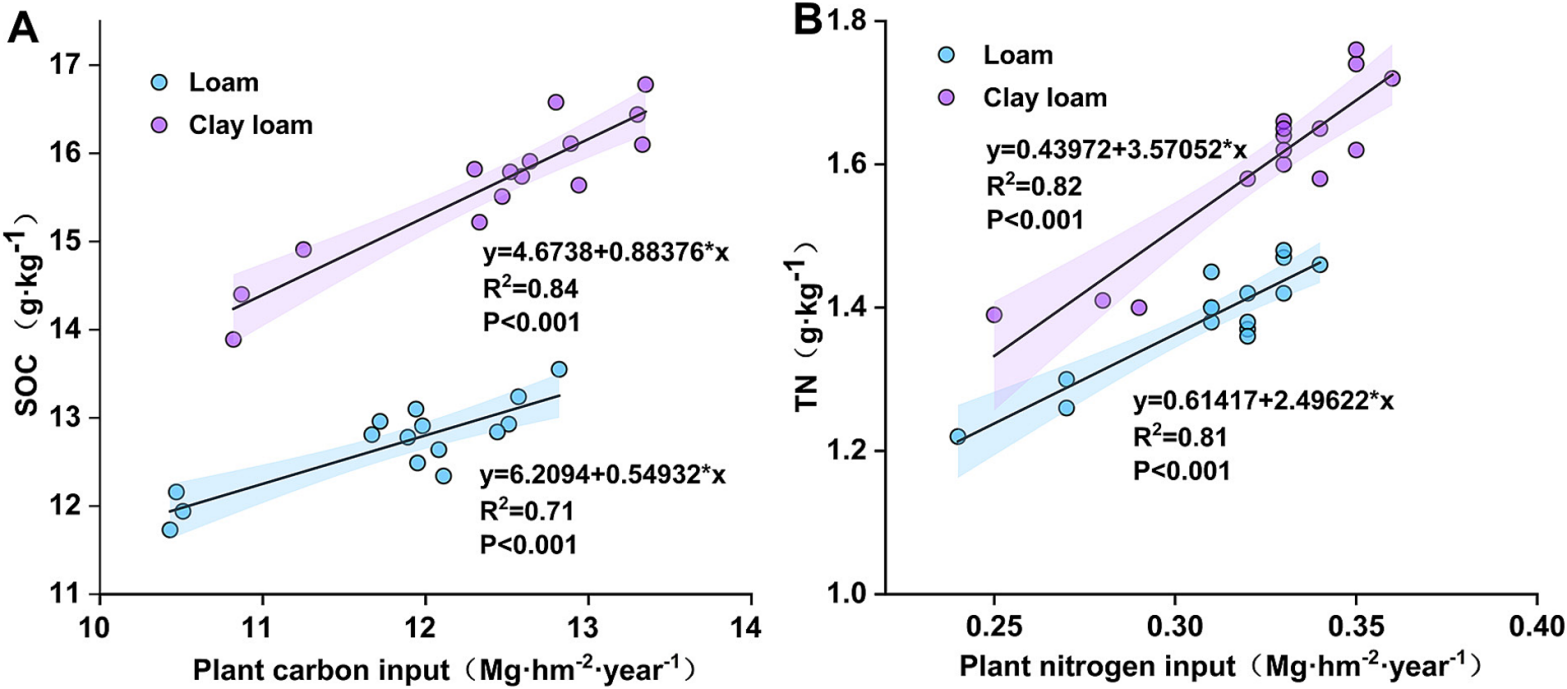
Linear regression between plant carbon and nitrogen inputs and SOC **(A)**, TN **(B)** after straw return.

After 50 days of straw burial, the highest SOC in the loam soil was observed under the N4:6 treatment, which was significantly higher than that under the N0 and N3:7 treatments at 190 days. In the clay loam soil, the N5:5 treatment consistently showed the highest SOC throughout the study period. At 20 days, SOC under the N5:5 treatment was significantly higher by 4.45–9.60% compared to N6:4 and N0 treatments. At 190 days, SOC under the N5:5 treatment was significantly higher than that under N0 and N3:7 treatments in 2020–2021. In 2021–2022, it was significantly higher than that under the N0, N4:6, and N6:4 treatments. At the wheat harvest stage, SOC in clay loam soil was higher than in loam soil, and N application treatments resulted in higher SOC than with no N application (*p* < 0.05). In the loam soil, the highest SOC was observed under the N4:6 treatment, while in the clay loam soil, it was observed under the N5:5 treatment. Correlation analysis indicated a significant positive correlation between SOC and plant carbon inputs (Figure 5).

### 3.4 TN

The results from the 2-year experiments (Table 3) indicated that the dynamics of soil TN concentrations were similar to those of SOC. Soil texture, N application treatments, and the interaction between soil texture and N application treatments significantly affected soil TN concentrations (except at 130 days of decomposition in 2020–2021).

**Table 3:** Effects of N fertilizer basal-to-top-dressing ratios on TN in loam and clay loam soils from 2020 to 2022 (g·kg^−1^).

Soil texture (T)	N application (*N*)	2020–2021							2021–2022						
		0d	20d	50d	130d	190d	210d	240d	0d	20d	50d	100d	140d	190d	220d
Loam	N0	1.13b	1.33c	1.33c	1.07b	1.51c	1.43c	1.09b	1.14b	1.06b	1.23b	1.12d	1.37b	1.42d	1.26d
	N3:7	1.21a	1.43ab	1.41b	1.38a	1.54bc	1.50bc	1.22ab	1.21ab	1.26a	1.35a	1.29b	1.52a	1.55b	1.37c
	N4:6	1.22a	1.48a	1.60a	1.40a	1.66a	1.62a	1.42a	1.27a	1.29a	1.37a	1.23c	1.53a	1.65a	1.47a
	N5:5	1.24a	1.40bc	1.48b	1.38a	1.52c	1.45c	1.15b	1.24a	1.24a	1.28b	1.25bc	1.52a	1.56b	1.40bc
	N6:4	1.18ab	1.47a	1.61a	1.45a	1.60ab	1.52b	1.18b	1.22ab	1.31a	1.39a	1.35a	1.49a	1.49c	1.42b
Clay loam	N0	1.28c	1.75b	1.54b	1.91b	1.90b	1.36d	1.25d	1.35c	1.56d	1.59b	1.53b	1.63c	1.51b	1.40d
	N3:7	1.38b	1.82b	1.63a	2.35a	2.14a	1.43cd	1.54b	1.39c	1.61c	1.64b	1.56b	1.78b	1.58b	1.65b
	N4:6	1.39b	1.84b	1.58ab	2.25a	2.02a	1.58bc	1.44c	1.46b	1.68ab	1.62b	1.61ab	1.90a	1.72a	1.60c
	N5:5	1.48a	2.08a	1.64a	2.40a	2.01a	1.77a	1.67a	1.55a	1.70a	1.72a	1.69a	1.96a	1.79a	1.74a
	N6:4	1.42b	1.79b	1.53b	2.25a	2.05a	1.70ab	1.53b	1.48b	1.66b	1.60b	1.67a	1.72bc	1.71a	1.62bc
Soil texture (T)		***	***	***	***	***	*	***	***	***	***	***	***	***	***
N application (N)		***	***	***	***	***	***	***	***	***	***	***	***	***	***
T × N		*	***	***	ns	**	***	**	*	**	***	*	**	***	***

Mean with ANOVA results (*n* = 3). Different lowercase letters in each column represent significant differences between different N treatments (*p* < 0.05; Duncan test). “T” and “N” represent the influences of soil texture and N treatments, respectively, based on two-way ANOVA. ns, *, **, and *** represent non-significant, *p* < 0.05, *p* < 0.01, and *p* < 0.001, respectively.

In the early stage of straw decomposition (0–50 days), TN in the loam soil was highest under the N6:4 ratio treatment, followed by the N4:6 ratio. At 50 days, the TN under the N6:4 treatment was significantly higher than under the N5:5 treatment, by 8.11–8.59%. In the clay loam soil, TN was highest under the N5:5 treatment, showing a significant increase of 12.79–18.59% compared to the other treatments at 20 days. In the later stages of straw decomposition (after 50 days), TN in loam soil peaked at 190 days of straw decomposition in both years. The N4:6 treatment showed a significantly higher TN concentration, by 5.77–9.21%, compared to the N3:7 and N5:5 treatments. In the clay loam soil, TN peaked at 130 days of straw decomposition in 2020–2021 and at 100 days in 2021–2022. At the wheat harvest stage, TN was higher in the clay loam soil than in the loam soil, and N application treatments resulted in higher TN than without N treatment. In the loam soil, the N4:6 ratio had the highest TN concentration; whereas, in the clay loam soil, the N5:5 treatment resulted in the highest TN concentration. Correlation analysis indicated a significant positive correlation between soil TN concentration and plant nitrogen input (Figure 5).

### 3.5 Soil bacterial diversity and composition

**Figure 6:**
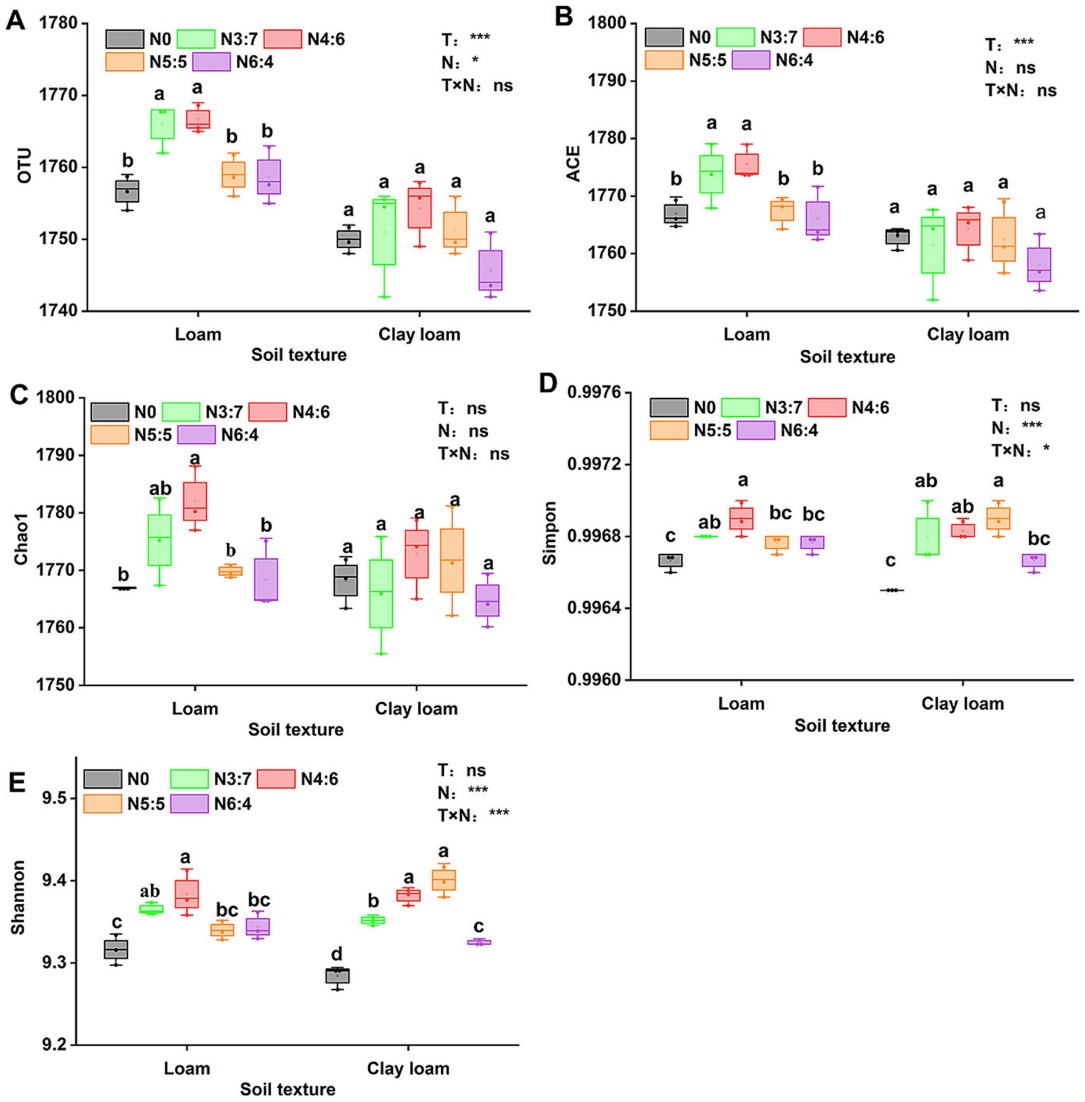
Effect of soil textures and N application treatments on soil bacterial alpha diversity, including OTU **(A)**, ACE **(B)**, Chao1 **(C)**, Simmpon **(D)**, and Shannon **(E)** indices. Different lowercase letters represent significant differences between different N treatments (*p* < 0.05; Duncan test). “T” and “N” represent the influences of soil texture and N treatments, respectively, based on two-way ANOVA. ns, *, **, and *** represent non-significant, *p* < 0.05, *p* < 0.01, and *p* < 0.001, respectively.

**Figure 7:**
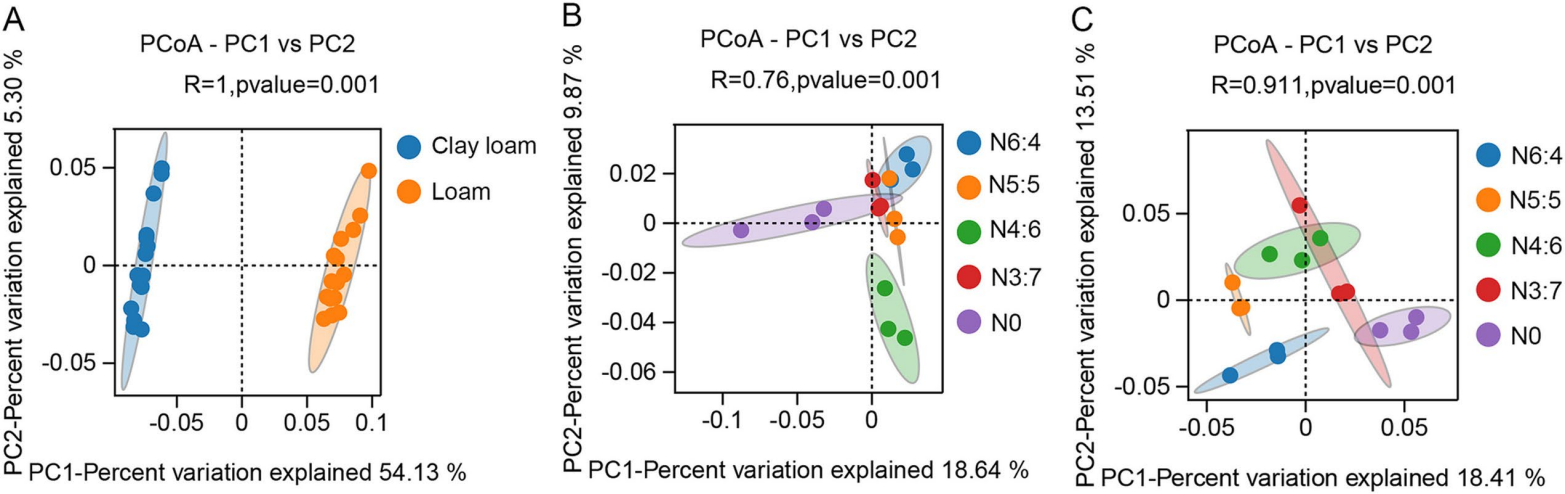
Effect of soil textures **(A)** and N application treatments in loam **(B)** and clay loam **(C)** on bacterial beta diversity. Each point in the figure represented a sample, different colors denoted different groups, and ellipses represented 95% confidence intervals. The x-axis represented the first principal coordinate, with the percentage indicating the contribution of the first principal coordinate to sample variation. The y-axis represented the second principal coordinate, with the percentage showing the contribution of the second principal coordinate to sample variation.

The bacterial alpha diversity (Figure 6) was evaluated using five common metrics (i.e., operational taxonomic units (OTUs) richness, and the Chao1, ACE, Simpson, and Shannon indices). Bacterial richness (OTU richness, Chao1, and ACE) in loam soil was significantly higher than in clay loam soil, whereas no significant difference in bacterial diversity (Simpson and Shannon) was observed between the two soil textures. In the loam soil, bacterial richness and diversity were highest under the N4:6 treatment. In the clay loam soil, bacterial diversity was the highest under the N5:5 treatment. Principal coordinate analysis (PCoA) revealed a clear separation in the bacterial community composition between the loam and clay loam soils (Figure 7). Specifically, in the loam soil, the N4:6 treatment showed a significant difference in bacterial community composition compared to the other treatments (Figure 7). In the clay loam soil, the bacterial community compositions under the N5:5 and N6:4 treatments were also notably different from those of the other treatments (Figure 7).

**Figure 8:**
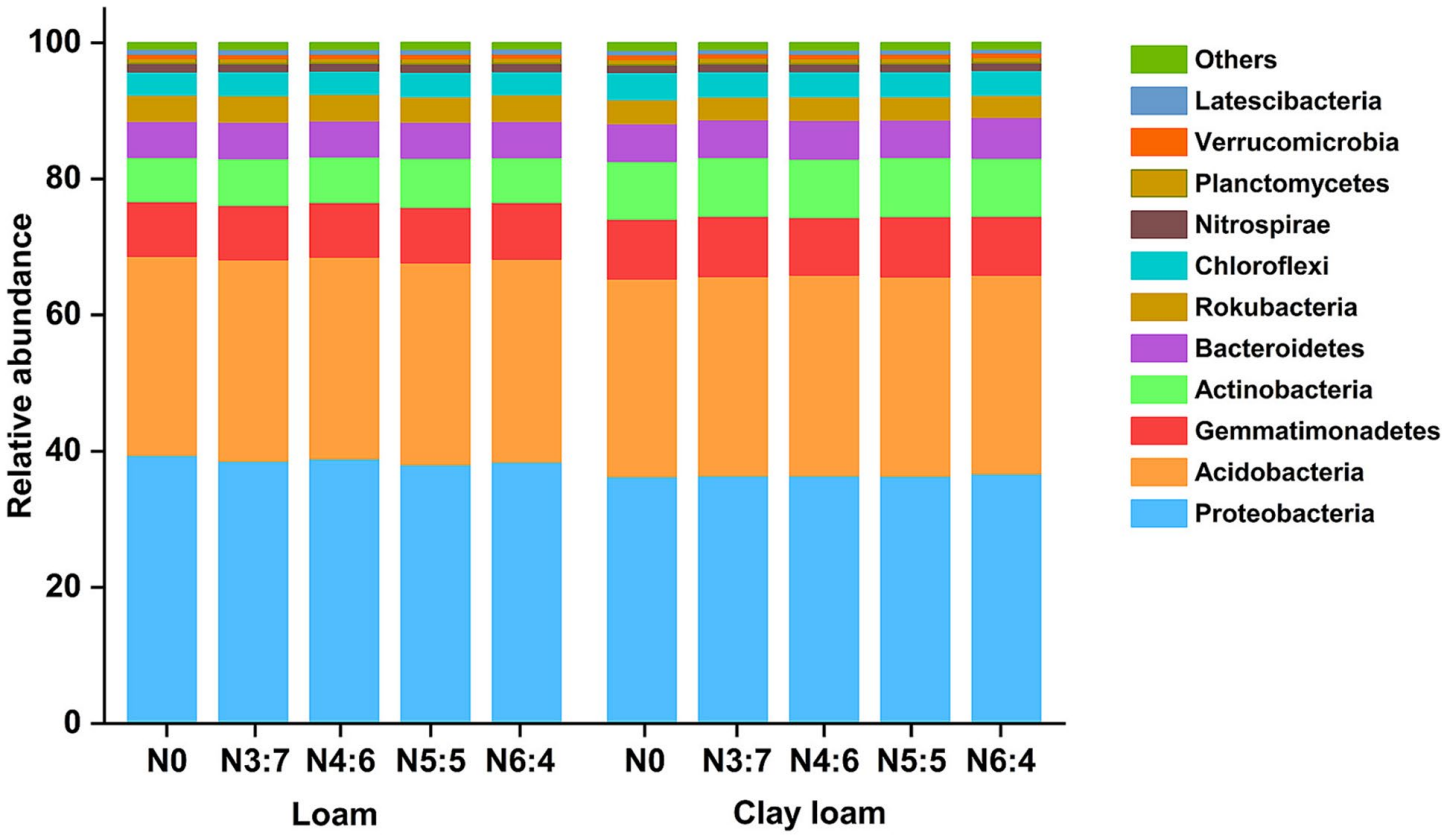
Effects of N treatments on soil bacterial community abundance at the phylum level in different soil textures.

**Figure 9:**
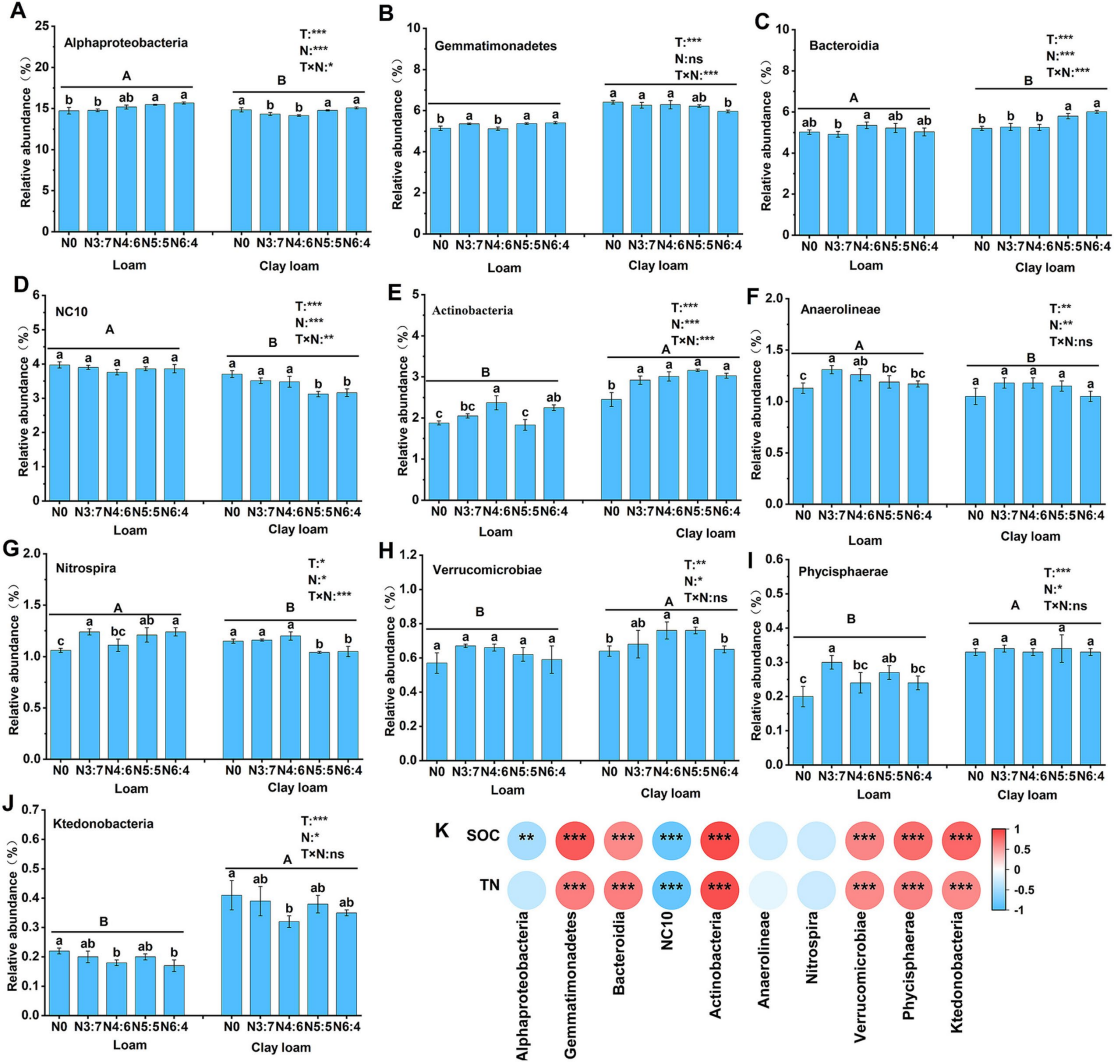
Effects of N treatments on soil bacterial community abundance at the class level in different soil textures **(A–J)**, and the correlation between these bacterial community abundances and SOC and TN **(K)**. Different lowercase letters represent significant differences between different N treatments, different capital letters represent significant differences between different soil textures (*p* < 0.05; Duncan test). “T” and “N” represent the influences of soil texture and N treatments, respectively, based on two-way ANOVA. ns, *, **, and *** represent non-significant, *p* < 0.05, *p* < 0.01, and *p* < 0.001, respectively.

In both the loam and clay loam soils, the dominant bacterial phyla were Proteobacteria, Acidobacteria, Gemmatimonadetes, Actinobacteria, Bacteroidetes, and Chloroflexi (Figure 8); however, their relative abundances varied. In the loam soil, the abundance of Proteobacteria was significantly higher, by 6.16%, than in clay loam soil. In contrast, the abundances of Gemmatimonadetes, Actinobacteria, and Bacteroidetes were significantly lower, by 7.38, 21.01, and 6.10%, respectively, in the loam soil compared to that in the clay loam soil. Different N application treatments had minimal impacts on bacterial relative abundances (>1%) at the phylum level but did alter the relative abundances of bacteria (>0.1%) associated with soil carbon and nitrogen turnover at the class level (Figures 8, 9).

At the class level, compared to the N0 treatment, N application increased the relative abundances of Bacteroidia, Anaerolineae, Actinobacteria, and Verrucomicrobia, with maximum increases of 15.58, 15.93, 28.98, and 18.75% (*p* < 0.05), respectively. In the loam soil, the relative abundance of Actinobacteria and Bacteroidia in the N4:6 treatment was significantly higher than that in the N3:7 treatment. In contrast, the relative abundance of Gemmatimonadetes, Nitrospira, and Phycisphaerae was significantly lower in the N4:6 treatment compared to the N3:7 treatment. In the clay loam soil, the relative abundance of Alphaproteobacteria and Bacteroidia in the N5:5 and N6:4 treatments was significantly higher than in the N3:7 and N4:6 treatments. Meanwhile, the relative abundance of NC10 and Nitrospira was significantly lower in the N5:5 and N6:4 treatments compared to the N3:7 and N4:6 treatments. Correlation analysis (Figure 9) indicated that SOC and TN were significantly positively correlated with Gemmatimonadetes, Bacteroidia, Actinobacteria, Verrucomicrobiae, Phycisphaerae, and Ktedonobacteria and were significantly negatively correlated with NC10.

### 3.6 Correlation analysis between indicators

**Figure 10:**
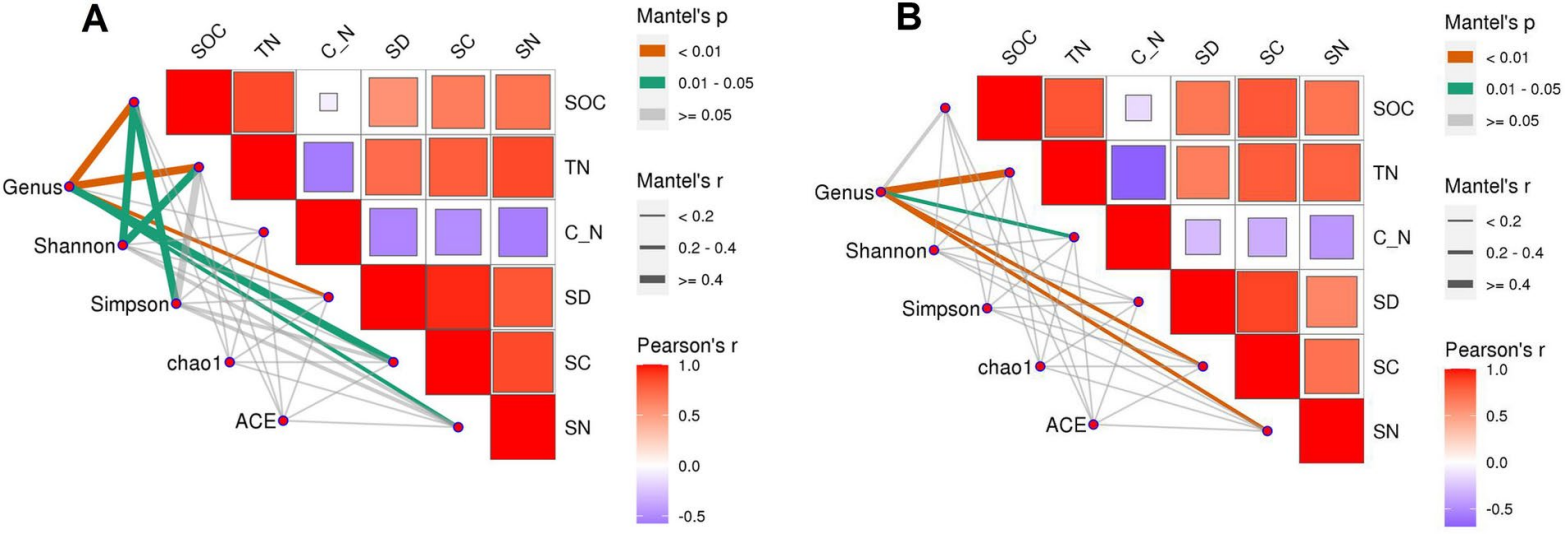
Correlation between straw decomposition, C and N release characteristics, and SOC, TN, and bacterial diversity in loam **(A)** and clay loam **(B)**. C_N: soil C/N ratio, SD: the cumulative decomposition rate of straw. SC: the cumulative C release rate of straw. SN: the cumulative N release rate of straw.

**Figure 11:**
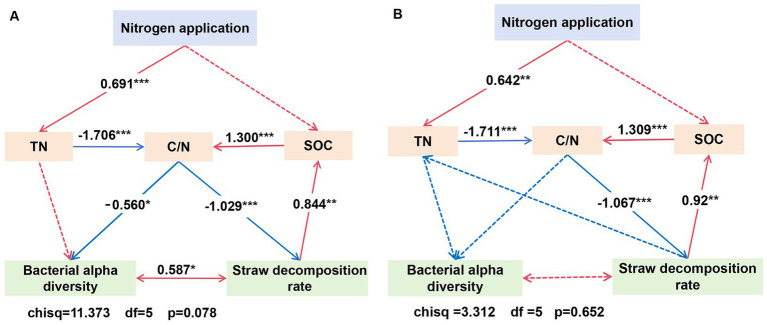
Structural equation model of the effect of N application treatments, SOC and TN, and bacterial alpha diversity on straw decomposition rates in loam **(A)** and clay loam **(B)**. Bacterial alpha diversity was assessed through dimensionality reduction using a principal component analysis (PCA) of the indices, including the ACE, Chao1, Simpson, and Shannon indices.

Correlation analysis between indicators showed that, in the loam soil, the Shannon index was significantly correlated with SOC and TN, and the Simpson index was significantly correlated with SOC (Figure 10). The results of the structural equation model (Figure 11) indicated that, in the loam soil, increasing the N fertilizer ratio enhanced maize straw decomposition by raising soil TN concentration and lowering the C/N ratio. This improvement in decomposition further contributed to an increase in both soil organic carbon concentration and soil bacterial diversity. In clay loam soil, increasing the nitrogen fertilizer ratio similarly promoted maize straw decomposition by raising soil TN concentration and lowering the C/N ratio. However, while this process resulted in higher SOC concentration, the relationship between maize straw decomposition and microbial diversity in this soil type was not significant.

## 4 Discussion

### 4.1 Effect of different N fertilizer basal-to-top-dressing ratios on maize straw decomposition and nutrient release

We found that straw decomposition rates increased rapidly within 50 days of returning to the soil, reaching over 40%, and then significantly decreased. In 2020–2021 and 2021–2022, decomposition rates reached 69.48–75.04% after straw was buried in the experimental soils for 240 and 220 days, respectively (Figure 2), which is consistent with previous research (Latifmanesh et al., 2020). This rapid initial decomposition is caused by the breakdown of soluble components in the straw through secondary metabolism. As easily decomposable materials are gradually consumed by microorganisms, the decomposition rate decreases. Eventually, decomposition ceases because the remaining components are difficult to degrade (Min et al., 2022). Straw decomposition is influenced by various factors, including straw types, the soil environment, and field management practices (Guan et al., 2020; Hassink, 1997; Henriksen and Breland, 2002; Min et al., 2022; Yadvinder-Singh et al., 2010). Studies have shown that the decomposition rate of crop residues varies among crop types, with maize straw decomposing faster than bean straw and wheat straw releasing more nitrogen and phosphorus nutrients than maize straw (Guan et al., 2020; Zhang et al., 2021). Hassink (1997) and Henriksen and Breland (2002) noted that the rate of straw decomposition in soil is negatively correlated with clay content because clay may bind to organic matter, altering the physical and biological environment for straw decomposition. Yadvinder-Singh et al. (2010) reported that straw decomposition rates in sandy soils were significantly higher than those in silty soils, although some studies have shown that soil texture has a minimal effect on straw decomposition and nutrient release (Yang et al., 2015).

Our study indicated that during the wheat season of 2020–2021, the decomposition rate of maize straw in clay loam soil was significantly higher than that in loam soil. However, during the wheat season of 2021–2022, during the early stage of straw decomposition (0–50 days), the loam soil had a higher decomposition rate than the clay loam soil, with minimal differences during the later stages of decomposition (50–220 days) (Figure 2). This variation may be attributed to the differences in rainfall; rainfall in October 2021 was significantly higher than that in October 2020, leading to a higher soil moisture content in 2021 during the early decomposition phase (0–50 days). Notably, the decomposition of maize straw is less constrained in finer-textured soils under dry conditions (Li et al., 2020).

N application also affects straw decomposition during maize growing. Some studies have reported that applying a certain ratio of nitrogen and phosphorus, along with timely nutrient replenishment, can accelerate straw transformation in the soil (Min et al., 2022; Zhu et al., 2018). In our study, over the entire wheat season, the decomposition rate of straw with N application was significantly higher than that without N application. Treatments N4:6 and N5:5 resulted in higher straw decomposition rates in both the loam and clay loam soils (Figure 2). Straw decomposition and nutrient release occurred simultaneously, and the patterns of straw C and N release were consistent with the dynamic changes in straw decomposition rates. During the wheat harvest stage, the C release rate (from 69.03 to 78.25%) was higher than that of N (from 58.25 to 68.35%). This is mainly because most of the nitrogen in straw exists in its organic form, as proteins, nucleic acids, chlorophyll, and other organic components in the cells, which increase the degree of bonding and hinder decomposition and release (Devêvre and Horwáth, 2000).

### 4.2 Effect of N fertilizer basal-to-top-dressing ratios on SOC and TN

After straw is returned to the soil, it decomposes under microbial action, increasing the SOC and TN content in various soil layers. However, the extent of this increase varies with factors such as the amount of straw added, the number of years of straw return, and field management practices (Lei et al., 2022; Liu et al., 2014; Mack et al., 2004; Wang et al., 2018; Yan et al., 2018). Our investigation revealed that the SOC and TN in both soil textures increased throughout the wheat-growing stage following straw return. SOC and TN were higher in the clay loam soil than in the loam soil, and were higher with N application than without N application (Tables 2, 3). A previous study identified silt and clay contents as the primary factors determining the maximum carbon and nitrogen accumulation in soil (Matus, 2021); clay loam soil exhibits better adsorption and retention of C and N released from straw, as its heavier texture hinders soil microorganisms and enzyme activity, resulting in slower decomposition and better preservation of SOC and nitrogen (Gentile et al., 2013; Mao et al., 2024). Additionally, our results indicate that plant carbon and nitrogen inputs were significantly and positively correlated with SOC and TN after straw return (Figure 5). A meta-analysis demonstrated that N addition increases terrestrial plant biomass by an average of 55.62%, thereby enhancing plant carbon and nitrogen inputs (Feng et al., 2023).

Throughout the wheat-growing season, the dynamic changes in SOC and TN exhibited notable differences. During straw decomposition, peak SOC values were observed at 20 and 190 days in both soil textures; at day 20, the highest SOC in the loam was found under the N6:4 treatment, while in the clay loam, the highest SOC was observed under the N5:5 treatment (Table 2). This may be attributed to the increase in microbial activity caused by higher N application, which accelerates the decomposition of maize straw and releases large amounts of nutrients (Figures 2, 3), leading to an increase in SOC (Latifmanesh et al., 2020; Min et al., 2022). Research has shown that N application leads to a reduction in soil C/N ratios. According to the “microbial N mining” hypothesis, microorganisms no longer need to decompose soil organic matter to obtain nitrogen. This could potentially alleviate the decomposition of existing SOC (Dijkstra et al., 2013). In clay loam soils, with their higher soil fertility, the initial N application likely stimulates microbial activity, promoting straw decomposition and SOC accumulation. However, excessive N application may lead to soil organic carbon mineralization, thereby reducing the organic carbon content (Bai et al., 2023).

At 190 days (the wheat booting stage in 2020–2021, and wheat grain-filling stage in 2021–2022), the N4:6 treatment had the highest SOC in the loam soil, while in the clay loam, this was observed under the N5:5 treatment (Table 2). This is mainly because a higher proportion of top-dressing in loam can enhance microbial activity, further promoting straw decomposition and increasing SOC. Additionally, N addition can stimulate plant growth and promote the accumulation of carbon in both aboveground and belowground plant residues, thereby increasing SOC (Schulte-Uebbing and de Vries, 2018; Xu et al., 2021). In clay loam, a balanced N fertilizer basal-to-top-dressing ratio of 5:5 can ensure adequate N availability for wheat growth while preventing the negative effects of excessive N, such as soil acidification and microbial imbalance, thereby promoting wheat growth and SOC accumulation.

We also observed that in loam, TN peaked at 50 and 190 days, while in clay loam, it peaked at 20 days and 130–140 days. However, the reason for this phenomenon remains unclear. During the early stages of straw decomposition (0–50 days), the trend of soil TN was similar to that of SOC. The superior aeration and drainage of loam facilitate nitrogen transformation and microbial activity, allowing nitrogen to be more efficiently absorbed by plants. As the N application rate increased, the TN in the loam soil also increased. The high water-retention capacity of clay results in slower N release. When N application rates are low, N can be effectively retained and released from clay loam. However, excessive N application may lead to a nitrogen surplus in clay, causing nitrogen volatilization, leaching, and nitrogen saturation effects, which reduce the soil TN concentrations (Liu C. R. et al., 2022; Ren et al., 2024). In the later stages of straw decomposition (after 50 days), the highest TN in the loam soil was observed under the N4:6 treatment, while in the clay loam, this was higher under the N5:5 treatment. This difference can be attributed to the fact that in loam soil, a higher top-dressing nitrogen ratio (N4:6) during the later stages of plant growth provides an additional nitrogen source. This stimulates the activity of fast-growing microbial communities, promotes nitrogen mineralization and transformation, and increases nitrogen availability, thus enhancing the TN concentration. The high water-retention capacity of clay loam results in slower nitrogen release. When the fertilization ratio is N5:5, the nitrogen supply is more balanced. This strategy helps maintain microbial community stability by preventing imbalances and excessive competition that can arise from the over-application of nitrogen. A balanced nitrogen supply allows microorganisms to more efficiently absorb and transform nitrogen while minimizing nitrogen losses (such as volatilization and leaching), thereby maintaining higher TN levels.

### 4.3 Effect of N fertilizer basal-to-top-dressing ratios on soil bacterial community diversity and abundance

Soil microbial communities play a crucial role in straw decomposition and soil nutrient cycling, and their community structure varies significantly with the straw return amount, soil properties, and management practices (Fan et al., 2019; Guo et al., 2020; Roberts et al., 2011; Xia et al., 2020). Xia et al. (2020) reported that soil texture was the second-largest factor affecting soil microbial community structure, followed by soil pH. The rupture of large pores also leads to higher bacterial richness in coarser-textured soils (Chau et al., 2011), and bacterial diversity in clay soils tends to be higher than that in sandy soils (Sessitsch et al., 2001). In our study, bacterial richness (Chao1, ACE, and OTU richness) in the loam soil was significantly higher than that in the clay loam soil, although bacterial diversity (Shannon and Simpson indices) between the two soil textures was similar (Figure 6). The differences in the results may be related to several factors, such as soil fertility and climatic conditions. Soil texture significantly affected the composition of soil bacterial communities, with previous studies showing that the relative abundances of Actinobacteria and Chloroflexi significantly increase with higher clay content, whereas *α*-Proteobacteria and Bacteroidetes are more abundant in coarser textured soils (Hemkemeyer et al., 2018; Karimi et al., 2018; Xia et al., 2020). We found that the abundance of Proteobacteria was significantly higher, by 6.16%, in the loam soil than in the clay loam soil, whereas the abundances of Gemmatimonadetes, Actinobacteria, and Chloroflexi were significantly lower, by 7.38, 21.01, and 7.36%, respectively (Figure 8).

In agricultural ecosystems, the metabolism of heterotrophic microorganisms is limited by soil nutrients such as C, N, and P (Wang et al., 2024). It has been reported that N application directly affects soil bacterial diversity and indirectly influences bacterial community abundance (Zeng et al., 2016). The application of N fertilizer after straw return can alleviate microbial N limitation and positively affect microbial diversity and abundance (Liu B. et al., 2022; Song et al., 2024). In our study, bacterial richness and diversity were highest under the N4:6 treatment in the loam soil, which were significantly higher than those under the other treatments. In the clay loam soil, the bacterial diversity under the N5:5 treatment was significantly higher than that under the N0 and N6:4 treatments (Figure 6). This phenomenon might be related to the distinct responses of soil textures to nitrogen release and microbial community dynamics. In loam soil, good aeration and low clay content allow nitrogen to release and be absorbed quickly (Thomsen et al., 2003). A higher top-dressing ratio (N4:6) provides sufficient nitrogen for wheat during its late growth stages, promotes wheat development, and accelerates maize straw decomposition. These processes raised SOC levels, enhancing microbial diversity (Liu H. Y. et al., 2023; Xu et al., 2021). In contrast, clay loam, with its higher clay content, releases nitrogen more slowly (Thomsen et al., 2003). Balanced fertilization (N5:5) reduces nitrogen loss, meets wheat’s nitrogen demands, and promotes maize straw decomposition, thereby increasing SOC concentration and maintaining microbial diversity (Esmaeilzadeh-Salestani et al., 2021). Our structural equation model further supported this explanation, demonstrating that optimizing the nitrogen fertilizer basal-to-top-dressing ratio could promote maize straw decomposition by increasing TN concentration and regulating the soil C/N rato, thereby enhancing SOC levels and microbial diversity. Straw return and N application significantly affect the relative abundances of microorganisms involved in soil carbon and nitrogen turnover (Ma et al., 2020). For example, Gemmatimonadetes, Bacteroidia, Actinobacteria, and Verrucomicrobiae primarily participate in the decomposition of complex organic matter and nutrient cycling, thereby influencing soil organic carbon mineralization and nitrogen release (Chen et al., 2024). The NC10 phylum primarily participates in denitrification in anaerobic environments, affects N loss and soil N availability, and indirectly influences soil carbon cycling (Bai et al., 2024). Our results indicated that N application increased the relative abundances of Bacteroidia, Anaerolineae, Actinobacteria, and Verrucomicrobiae compared to no N application (Figure 9). This likely occurred because N fertilization enhanced the N supply in the soil, alleviated N limitation, and increased the metabolic activity of these microbial phyla (Song et al., 2024). In the loam soil, the N4:6 treatment exhibited the highest relative abundance of Bacteroidia and Actinobacteria; whereas in the clay loam soil, the N5:5 and N6:4 treatments showed higher relative abundances of these phyla. This was likely due to the higher straw decomposition rates under the N4:6 and N5:5 treatments, in both soil textures, which provided ample carbon sources to support the proliferation of Bacteroidia and Actinobacteria. Our results also confirm that SOC and TN are significantly positively correlated with Gemmatimonadetes, Bacteroidia, Actinobacteria, Verrucomicrobiae, Phycisphaerae, and Ktedonobacteria, and significantly negatively correlated with NC10 (Figure 9).

### 4.4 Optimizing N fertilizer basal-to-top-dressing ratios for sustainable agriculture: ecological and economic implications

The majority of studies to date have demonstrated that optimizing N application ratios enhances crop photosynthesis, improves nitrogen use efficiency, reduces greenhouse gas emissions, and increases crop yields (Wang et al., 2023; Zhang et al., 2020; Zhang et al., 2022). This study highlights that tailoring the basal-to-top-dressing nitrogen ratio based on soil texture in wheat–maize rotation systems significantly enhances SOC concentrations and microbial diversity. In loam soil, a higher top-dressing ratio (N4:6) accelerated maize straw decomposition, boosted carbon inputs, and enhanced microbial diversity. Conversely, in clay loam soil, a balanced nitrogen ratio (N5:5) supported microbial community stability and maintained soil fertility. These improvements contribute to enhanced soil quality, increased agricultural productivity, and progress toward sustainable agricultural development. Additionally, this study integrated straw return with optimized N application ratios, which can reduce the reliance on chemical inputs while increasing SOC concentrations. These approaches are particularly well suited for agroecosystems aiming for sustainable intensification, especially in the North China region. Economically, the proposed fertilization strategy is both feasible and advantageous. Adjusting the basal-to-top-dressing nitrogen ratio incurs minimal costs and does not require additional fertilizer inputs or specialized equipment. Previous research from our studies demonstrated that, compared to traditional fertilization methods, this strategy significantly increases wheat yields by 6.90–14.58% (Yuan et al., 2020; Zhang et al., 2022). In summary, this study presents both ecological and economic pathways for enhancing soil health and supporting sustainable agriculture. The majority of studies indicate that long-term fertilization (amount, types, and methods) can significantly increase SOC content and microbial diversity (Abdalla et al., 2022; Dai et al., 2018; Luo et al., 2023; Ma et al., 2022; Tian et al., 2015; Xu et al., 2021). However, the long-term impacts of these nitrogen ratios on soil quality and microbial communities merit further investigation. We hypothesize that long-term application of fertilizers with these nitrogen ratios could sustain soil quality and promote microbial community stability. However, long-term monitoring is required to validate this hypothesis.

## 5 Conclusion

The optimization of N fertilizer basal-to-top-dressing ratios enhanced SOC and TN by accelerating maize straw decomposition and nutrient release, as well as by increasing plant carbon and nitrogen inputs. Among the treatments, the N4:6 ratio in loam and the N5:5 ratio in clay loam were found to be the most effective. Furthermore, SOC and TN were significantly positively correlated with Gemmatimonadetes, Bacteroidia, Actinobacteria, Verrucomicrobiae, Phycisphaerae, and Ktedonobacteria, while being significantly negatively correlated with NC10. The N4:6 treatment in loam and N5:5 treatment in clay loam exhibited higher bacterial richness and diversity. Therefore, during the wheat-growing season, N fertilizer ratios of 4:6 in loam and 5:5 in clay loam can accelerate straw decomposition and C and N release, enhance SOC and TN, improve soil microbial diversity and abundance, optimize straw resource utilization, and promote healthy and sustainable soil development. Future research should evaluate the long-term effects of nitrogen fertilizer strategies on soil health and productivity across diverse soil types and climatic conditions. Sustained efforts are crucial to developing strategies that balance agricultural productivity with environmental sustainability.

## Data Availability

The datasets presented in this study can be found in online repositories. The names of the repository/repositories and accession number(s) can be found at: https://www.ncbi.nlm.nih.gov/, PRJNA1150509.

## Footnotes

^1^ https://www.biocloud.net
